# Unexpected Electroencephalogram Activity in a Brain-Dead Organ Donor: A Case Report

**DOI:** 10.7759/cureus.96488

**Published:** 2025-11-10

**Authors:** Marta Afonso, Mariana Thedim, Liliana Dias, Ana Isabel Pereira, Pedro Amorim

**Affiliations:** 1 Department of Anesthesiology, Unidade Local de Saúde de Gaia/Espinho, Vila Nova de Gaia, PRT; 2 Department of Anesthesiology, Mass General Brigham, Boston, USA; 3 Department of Anesthesiology, Unidade Local de Saúde de Santo António, Porto, PRT

**Keywords:** bispectral index monitoring, brain death critical care, electroencephalography, neurology and critical care, physiologic monitoring

## Abstract

Brain death (BD) is the complete and irreversible loss of brain function, diagnosed primarily through clinical criteria. Electroencephalography (EEG) is a non-invasive tool commonly used to support the diagnosis by showing electrocerebral inactivity. The bispectral index (BIS), a processed EEG parameter, has been proposed as a potential adjunct for the early detection of neurological deterioration and BD. This case provides new insights into BIS monitoring in a brain-dead patient undergoing organ procurement surgery. A 56-year-old woman underwent organ procurement surgery following confirmed BD by clinical and ancillary criteria. Her history included obesity, hypertension, and dyslipidemia. She had suffered an out-of-hospital cardiac arrest with prolonged resuscitation, and no reversible cause was found. Imaging revealed severe cerebral edema and hypoxic-ischemic injury. EEG at 48 hours showed complete electrocerebral silence, and computed tomography angiography of the brain confirmed the absence of intracranial blood flow. Intraoperatively, standard American Society of Anesthesiologists monitoring and BIS™ were used. Hemodynamic stability was maintained with norepinephrine infusion, targeting a mean arterial pressure above 65 mmHg to preserve optimal organ perfusion. Normothermia and lung protective ventilation were ensured. Initially, BIS values remained around 50 with a suppression ratio ranging between 15 and 25, and no electromyographic activity was detected. EEG showed low-amplitude slow-wave activity. Following aortic cross-clamping and circulatory arrest, BIS dropped to zero, and the EEG trace remained isoelectric until the end of the case. Throughout the procedure, the patient remained hemodynamically stable. The surgery was uneventful, and the kidneys, liver, and corneas were successfully retrieved. In this case report, BIS monitoring was used to explore intraoperative EEG patterns in a patient with confirmed BD. BIS values remained around 50 during the procedure, dropping abruptly to zero only after circulatory arrest. As a simple, non-invasive tool, BIS has been proposed as an adjunct for neurologic monitoring in intensive care unit patients with declining values correlating with worsening neurological status and ultimately reaching zero at BD. This has led to the hypothesis that BIS could help detect early cortical inactivity, enabling timely BD diagnosis and ancillary testing, potentially expediting diagnosis and optimizing organ preservation. However, in this case, BIS values compatible with an anesthetized state persisted despite formal BD confirmation, which was consistent with previous reports. BIS readings can be affected by extracranial artifacts such as electrocardiographic activity, arterial pulsation, ballistocardiographic effects, electrocautery, patient handling, and electromyography. In this case, elevated BIS values likely reflected residual signals or artifacts rather than cortical activity. These findings emphasize that, while BIS can aid continuous monitoring and early detection of neurological deterioration, its interpretation in BD requires caution, with careful consideration of potential artifacts and physiological factors. BIS monitoring may support timely BD assessment in comatose patients, but its limitations and the influence of artifacts and physiological factors on BIS readings must be carefully considered.

## Introduction

Brain death (BD) is defined as the complete and permanent loss of brain function. Its diagnosis is primarily clinical, and it is based on three essential findings: unresponsive coma, absence of spontaneous respiration, and brainstem areflexia [1,2]. Ancillary tests, such as cerebral blood flow or electrophysiological studies, are indicated when the clinical exam cannot be completed, confounding factors persist, or when required by law [1]. For instance, electroencephalography (EEG) is a safe, non-invasive tool, frequently used to support the diagnosis of BD by demonstrating electrocerebral inactivity [1,3]. However, it requires complex multi-channel recording. In this context, the Bispectral Index (BIS) offers a simpler and more accessible complement to electrophysiological assessments, though its role in BD remains uncertain and is further explored in the present case.

BIS is an algorithm that processes frontal EEG data from a single channel into a numerical index reflecting the patient’s level of hypnosis. This simplified and real-time index ranges from 100 (fully awake) to zero (isoelectric EEG) [4]. BIS is used not only in the operating room (OR) to tailor anesthetic depth and reduce the incidence of intraoperative awareness, but also in Intensive Care Units (ICU) to monitor sedation depth in critically ill patients [4,5]. Furthermore, BIS has been proposed as a potential adjunct for early detection of severe neurologic deterioration and BD onset [6-11]. However, the strengths and limitations of BIS monitoring in the context of BD diagnosis remain to be explored.

The following case unravels new insights into BIS monitoring on a brain-dead patient undergoing organ procurement surgery, highlighting the need to consider artifacts and physiological factors when interpreting BIS values in the context of BD.

## Case presentation

**Figure 1:**
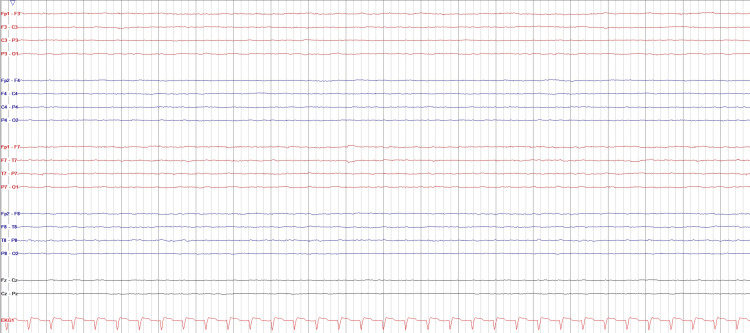
Twenty-second electroencephalogram segment recorded 48 hours after intensive care unit admission, showing electrocerebral silence (filters 0.5–70 Hz, sensitivity 7 µV/mm, notch 50 Hz).

A 56-year-old woman was admitted to the OR for organ procurement surgery following a confirmed diagnosis of BD, established through both clinical and ancillary testing. Her medical history included obesity, dyslipidemia, and arterial hypertension. She had previously been independent in daily activities and had no known allergies. Four days prior, she was admitted to the ICU following an out-of-hospital cardiac arrest. The collapse was sudden without any preceding symptoms, and emergency services were activated immediately. The patient was initially found in ventricular fibrillation, with an estimated 10 minutes of no-flow and 40 minutes of low-flow before return of spontaneous circulation was achieved. No definitive cause for the arrest was identified. Initial cranial computed tomography (CT) revealed severe diffuse cerebral edema. Despite full invasive support in the ICU, her neurological prognosis was poor. At 48 hours post-admission, the EEG demonstrated complete electrocerebral inactivity, and sedative drug levels were not sufficient to account for the findings (Figure 1). A repeat cranial CT scan confirmed diffuse and severe hypoxic-ischemic injury. On the third day of hospitalization, the patient developed clinical signs of cerebral herniation, and a BD evaluation was initiated. In accordance with Portuguese legislation, clinical testing included confirmation of unresponsive coma, absence of brainstem reflexes, and a positive apnea test [2]. Ancillary testing with CT angiography (CTA) demonstrated the absence of intracranial cerebral blood flow with preserved extracranial carotid circulation, findings consistent with BD. A second complete clinical examination was performed 12 hours later, confirming the diagnosis. The death was declared, and the family was informed. The patient remained on full invasive organ support until transfer to the OR for multiorgan procurement.

**Figure 2:**
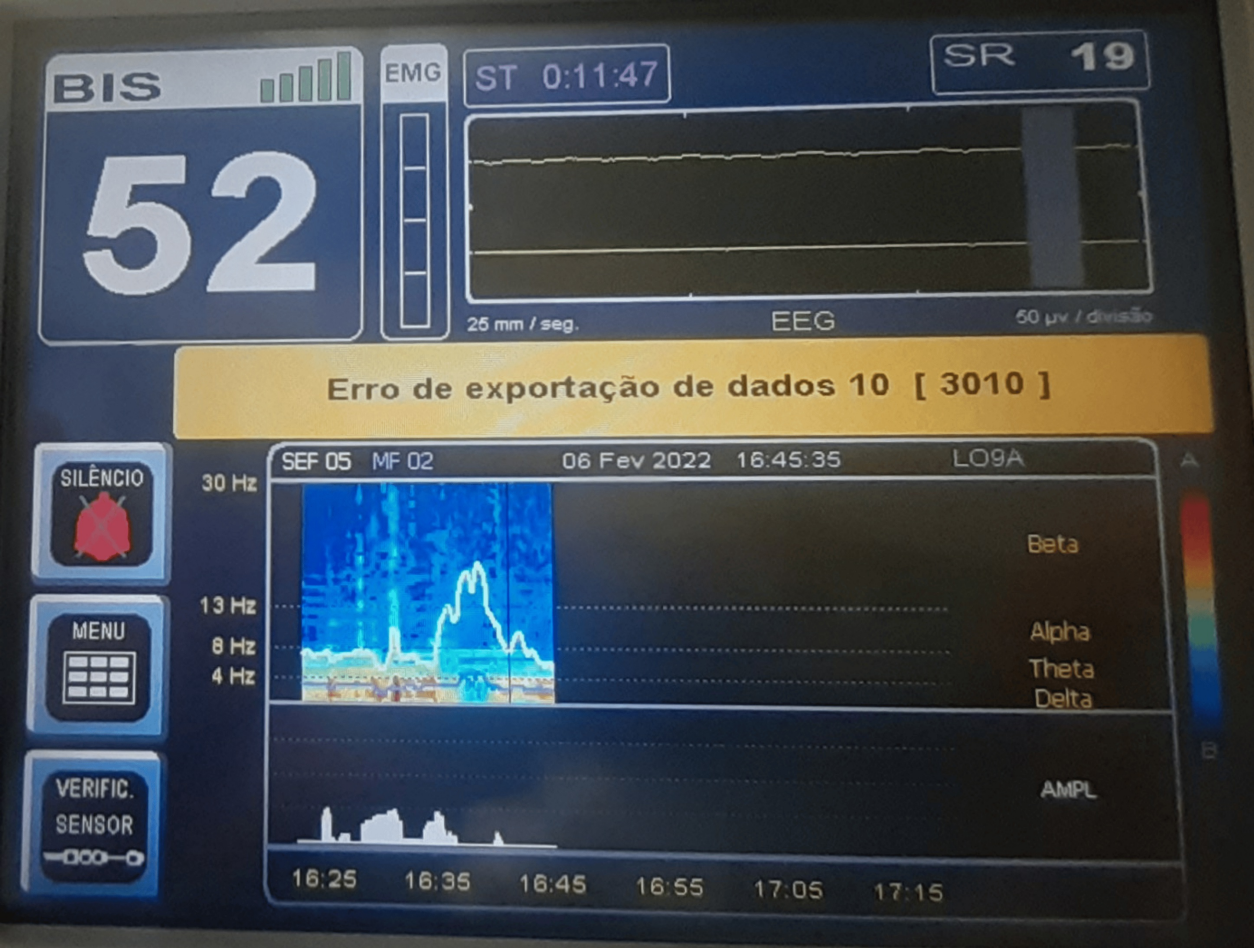
Electroencephalography trace as displayed on the Bispectral Index monitor before circulatory arrest. The yellow banner displays a warning message in Portuguese "Erro de exportação de dados" (translation: data export error).

**Figure 3:**
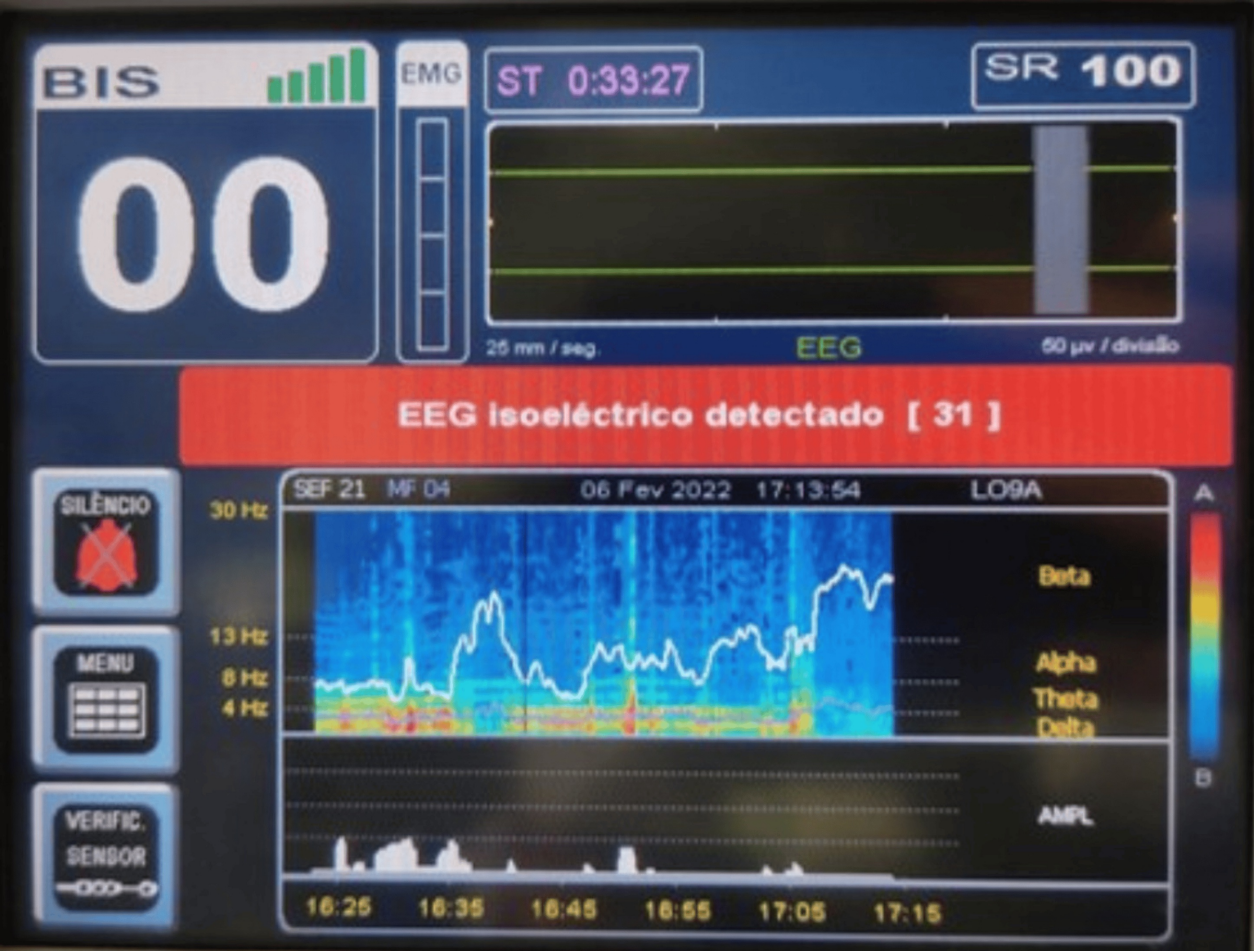
Isoelectric electroencephalography as displayed on the Bispectral Index monitor following circulatory arrest. The red banner displays a warning message in Portuguese "EEG isolelétrico detectado" (translation: Isoelectrical EEG detected).

Intraoperatively, standard American Society of Anesthesiologists (ASA) monitoring was applied with continuous electrocardiography (ECG), invasive arterial blood pressure, pulse oximetry, temperature, and urine output. A Medtronic BIS™ Complete 2-Channel Monitor was used to assess potential cortical electrical activity. Arterial blood gas monitoring included pH, electrolyte levels, lactate, hemoglobin, and blood glucose. To preserve optimal organ perfusion, hemodynamic stability was maintained with norepinephrine infusion targeting a mean arterial pressure above 65 mmHg. Lung protective ventilation was maintained, and fluids were administered according to goal-directed therapy. A forced-air warming device and fluid warmer were used to maintain normothermia. A bolus of 150 mcg fentanyl was administered to blunt any potential autonomic response to surgical incision, and 60mg rocuronium was given to eliminate any possible spinal reflex movements. Once BIS monitoring was initiated, its values remained around 50 with a good signal quality and no electromyographic interference (Figure 2). The EEG trace displayed irregular, very low-amplitude fluctuations mimicking residual cortical activity, although no true cerebral activity was present, as BD had been previously established. Frequency analysis revealed persistent activity in the delta and theta frequency bands, consistent with a slow-wave EEG pattern. The suppression ratio (SR) ranged between 15 and 25, indicating intermittent periods of electrocerebral inactivity. No external stimuli, besides the normal intraoperative procedures, occurred. Notably, shortly after aortic cross-clamping and circulatory arrest, BIS abruptly dropped to zero, and the EEG trace remained isoelectric until the end of the case (Figure 3). Electromyographic activity remained absent. In the final minutes of monitoring, following cessation of circulation, the spectrogram showed no detectable activity across any EEG frequency bands, with a uniform blue display. The SR reached 100, which indicates a completely suppressed EEG over the preceding 63 seconds.

The patient remained hemodynamically stable throughout the procedure, with adequate gas exchange, preserved urine output, and stable electrolyte and acid-base status. The organ procurement was completed without complications, successfully retrieving both kidneys, the liver, and the corneas.

Informed consent for publication of this case report was obtained from the patient’s family.

## Discussion

In this clinical case report, BIS monitoring was used as an exploratory tool to assess intraoperative EEG patterns in a patient with a confirmed diagnosis of BD. Interestingly, we found that BIS values remained around 50 throughout the procedure, only dropping abruptly to zero after circulatory arrest.

BIS monitoring is simple, continuous, non-invasive, and easy to interpret. It has been described as a potential adjunct in neurologic monitoring in ICU patients with brain injury, showing correlations with sedation scales and neurologic status in both sedated and non-sedated patients [6]. Several studies have documented BIS values in patients with severe neurological injury upon ICU admission [7-13]. As neurological status deteriorates, BIS values were found to progressively decline with a simultaneous increase in SR. Ultimately, the BIS values would reach zero, and the SR 100, which coincided with the diagnosis of BD [7-10]. In other studies, BIS monitoring was performed after BD diagnosis had been confirmed, with most patients exhibiting a BIS of zero [11-13]. These articles describe this phenomenon across different age groups, including in children, and different clinical settings, most frequently in cases of severe traumatic brain injury (TBI) [7-13]. Moreover, previous research reported a correlation between BIS values and neurologic outcomes in patients with refractory cardiac arrest treated with extracorporeal cardiopulmonary resuscitation and mild therapeutic hypothermia. These findings suggest that BIS may be useful both for predicting neurologic prognosis and for early detection of BD in this population [14]. Additionally, low BIS values have been associated with worse neurological outcomes in patients with TBI and post-cardiac arrest, supporting its potential prognostic role [15,16]. In line with this, BIS could be useful in identifying, intraoperatively, patients with severe irreversible brain injury [12].

BIS monitoring has been hypothesized to be a useful tool for capturing early signs of cortical inactivity leading to the timely BD diagnosis and subsequent deployment of ancillary testing [7-14]. Ultimately, this would expedite BD diagnosis with a potential optimization of organ preservation. However, in this case report, BIS values compatible with an anesthetized state persisted despite a formal diagnosis of BD supported by a 21-channel EEG showing complete electrocerebral inactivity. Similar findings have been reported previously, where patients with BD showed BIS values above 30 [11,12]. Although the BIS algorithm is derived from EEG activity, several factors may not be accounted for in its equation, leading to potentially misleading values in BD patients. For example, artifacts from extracranial sources, such as ECG activity, arterial pulsation, ballistocardiographic effects, electrocautery, patient handling, and EMG, can all contribute to artificially increasing BIS [11,12,17]. In our case, since EMG signals were prevented by rocuronium, these elevated BIS values can potentially reflect residual signals or artifacts that ceased entirely with the termination of systemic perfusion. Therefore, we hypothesize that ECG signals, temporal artery pulsation, or possibly ballistocardiographic signals may have been detected and misinterpreted as cerebral activity by the algorithm.

These observations highlight that BIS, although potentially useful for continuous monitoring and early warning of neurological deterioration, should be interpreted with caution in the setting of BD. Potential sources of artifact and physiological factors that may influence BIS values must be carefully considered. Due to the inherent limitations of relying solely on BIS indices, clinicians should also visually analyze raw EEG elements to ensure a cautious interpretation of the patient's brain status. In this case, intraoperative variables were continuously recorded but only registered in key moments of the surgical procedure. Future studies assessing BIS in BD should include continuous registration of intraoperative variables, including EEG-related patterns, to facilitate a better understanding of their temporal dynamics.

## Conclusions

As BIS provides a real-time and objective assessment of cortical electrical activity, it can help identify the transition to electrocerebral inactivity and BD onset. Thus, BIS monitoring can be useful for the timely deployment of ancillary BD testing in comatose patients. Nevertheless, its potential contribution to support a formal BD diagnosis can be compromised by several artifacts, residual drug effects, and hypothermia. Therefore, BIS should be used with caution in the confirmation of BD, and its interpretation must be integrated with standard clinical and ancillary assessments. This case underscores the limitations of BIS monitoring in BD patients and the importance of recognizing potential artifacts that may affect its algorithm.
